# A Joint Classification Method for COVID-19 Lesions Based on Deep Learning and Radiomics

**DOI:** 10.3390/tomography10090109

**Published:** 2024-09-05

**Authors:** Guoxiang Ma, Kai Wang, Ting Zeng, Bin Sun, Liping Yang

**Affiliations:** 1 School of Public Health, Xinjiang Medical University, Urumuqi 830017, China; xjmu_mgx@163.com (G.M.);; 2 College of Medical Engineering and Technology, Xinjiang Medical University, Urumuqi 830017, China

**Keywords:** radiomics, machine learning, classification, deep learning

## Abstract

Pneumonia caused by novel coronavirus is an acute respiratory infectious disease. Its rapid spread in a short period of time has brought great challenges for global public health. The use of deep learning and radiomics methods can effectively distinguish the subtypes of lung diseases, provide better clinical prognosis accuracy, and assist clinicians, enabling them to adjust the clinical management level in time. The main goal of this study is to verify the performance of deep learning and radiomics methods in the classification of COVID-19 lesions and reveal the image characteristics of COVID-19 lung disease. An MFPN neural network model was proposed to extract the depth features of lesions, and six machine-learning methods were used to compare the classification performance of deep features, key radiomics features and combined features for COVID-19 lung lesions. The results show that in the COVID-19 image classification task, the classification method combining radiomics and deep features can achieve good classification results and has certain clinical application value.

## 1. Introduction

Respiratory infectious diseases have become a major public health problem, a situation which has brought great challenges to global medical and health services. Therefore, early prevention, diagnosis and treatment are particularly important for improving the prognoses of patients, reducing the economic burden of patients, and avoiding the waste of medical resources [1]. Pneumonia caused by novel coronavirus infection is an acute respiratory infectious disease, common signs of which include respiratory symptoms, fever, cough, shortness of breath and other flu-like symptoms. When the disease worsens, it affects multiple tissues and organs, causes pneumonia and rapidly turns into severe acute respiratory syndrome and renal failure. The prognosis has a great impact on human organs and their functions [2]. Therefore, timely, accurate and effective diagnosis is the key to the treatment and prevention of infectious diseases such as SARS. At present, real-time Reverse Transcription–Polymerase Chain Reaction (RT-PCR) [3] of viral nucleic acid is the recommended method for the diagnosis of COVID-19 [4,5]. However, with the rapid increase in the number of infections, RT-PCR testing may become unreliable because of the viral load or sampling technique. In addition, RT-PCR relies heavily on manual sampling and has strict limitations on sampling criteria. A number of studies have shown the effectiveness of chest CT in the diagnosis of COVID-19, a technique in which deep learning and radiomics methods play important roles in the image-assisted diagnosis of lung diseases [6,7,8].

Common lung diseases include pneumonia, chronic obstructive pulmonary disease, bronchial asthma, pulmonary nodules, lung cancer, etc., and are typically accompanied by symptoms of cough, chest pain, fever, dyspnea, expectoration, hemoptysis and acute respiratory distress syndrome. Compared with other diseases, unknown viral lung diseases are usually highly contagious and may cause large-scale mass infection events in the short term, posing a certain threat to public healthcare [9]. Therefore, in the future, using medical imaging and clinical indicators to explore the characteristics of complex lung diseases can provide prevention methods for the occurrence of lung diseases which are more rapid and accurate. Microscopic changes in gene or protein patterns will be reflected in macroscopic images, and mining deep image features can reflect changes in human tissues, cells and genes. Deep learning and radiomics can extract a large amount of high-dimensional image information which can analyze disease information objectively and comprehensively, and thereby play a potential role in promoting disease diagnosis, treatment selection and prognosis evaluation [10].

Current research has shown that lung-image processing methods based on deep learning and radiomics can effectively distinguish lung disease subtypes, provide a better clinical prognosis accuracy and assist clinicians in adjusting clinical management level in time, allocating medical resources more reasonably [11]. Deep learning can automatically learn from a large amount of data and obtain the deep feature expression in the data, and it also has good feature-discrimination ability [12]. It can effectively improve the performance of machine-learning tasks, and has been widely used in signal processing, computer vision, natural language processing and many other fields [13,14,15]. In the medical field, Zhang et al. applied a deep neural network to the imaging-based diagnosis of COVID-19, which provided an important basis for the standardized diagnosis of COVID-19 [16]. In addition, good progress has been made in brain tumor MRI imaging-based diagnosis [17], pancreatic disease diagnosis [18], breast disease diagnosis [19], etc., each of which has provided new standardized diagnosis and treatment methods for hospitals. Radiomics can extract high-dimensional image features from conventional computed-tomography images and describe the imaging differences of human tissues and organs, thereby quantitatively assessing the disease and exploring the imaging markers. At present, it has been applied in many fields, such as tumor detection, lymphatic cancer metastasis prediction and treatment response evaluation [20,21,22]. In the diagnosis of lung diseases, radiomics has been shown to play an important role in COVID-19 screening, diagnosis, and prediction of hospital stay, as well as the assessment of risk factors related to pneumonia patients [23,24,25].

The use of deep learning and radiomics methods to automate the diagnosis of lung lesion subtypes can not only explore the individual differences in the imaging phenotypes of lung lesions, but also provide automated auxiliary diagnostic tools for clinical diagnosis in order to provide a better screening method for the prevention and control of infectious lung diseases. In this study, six machine-learning methods were used to compare the diagnostic efficacy of deep features, key radiomics features and combined features for different COVID-19 lung lesions. Radiomics features were used to analyze the differences in the imaging phenotypes of the different lung lesions, which provided a reference for clinical diagnosis of lung lesions. Through the study, we compared the performance of different modeling methods for lung disease classification, determined the radiomics features of different lung lesions and found a tool that can automatically identify lung lesions.

## 2. Materials and Methods

**Figure 1:**
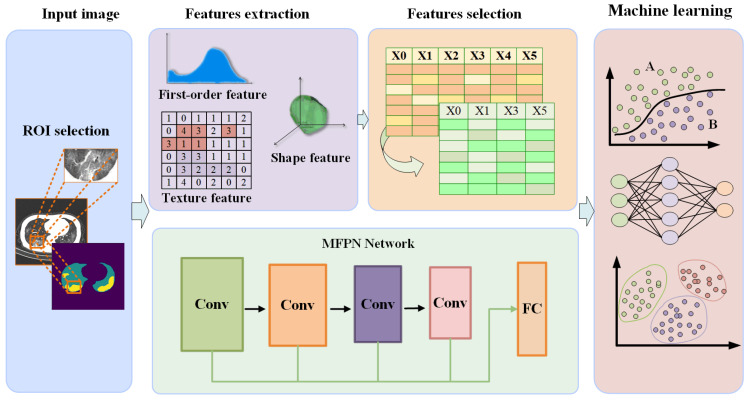
Technical framework diagram of a joint approach for deep learning and radiomics.

In this study, Python (version 3.5.6) was used to write the experimental code. The validation set was used for model evaluation, the ratio of training set and validation set was 4:1 and the average size of the lesion image was 66 × 66 pixels. The experimental process mainly includes image input, feature extraction, feature selection and machine-learning modeling; the flow chart is shown in Figure 1. Feature extraction mainly includes lesion-image radiomics feature extraction and depth feature extraction, A new multi-scale convolutional neural network was used to extract the depth features of lung lesions, and six machine-learning classifiers were used for the final classification task.

### 2.1. Data

The data used in this study are lung-CT images. The dataset is from China National Center for Bioinformation, via the China Consortium of Chest CT Image Investigation (CC-CCII) [26], which is publicly available globally and was compiled based on data from the Third Affiliated Hospital of Sun Yat-sen University, the First Affiliated Hospital of Anhui Medical University, the Huaxi Hospital of Sichuan University, the People’s Hospital of Jiangsu Province, the Central People’s Hospital of Yichang, and the People’s Hospital of Wuhan University. All hospitals obtained Institutional Review Board (IRB) or Independent Ethics Committee (IEC) review approval and the informed consent of subjects, and CC-CCII complied with the policy of the Chinese Center for Disease Control and Prevention as to reportable infectious diseases, the Chinese Health Quarantine Law and the Chinese patient privacy regulations, and also followed the principles of the Declaration of Helsinki.

**Figure 2:**
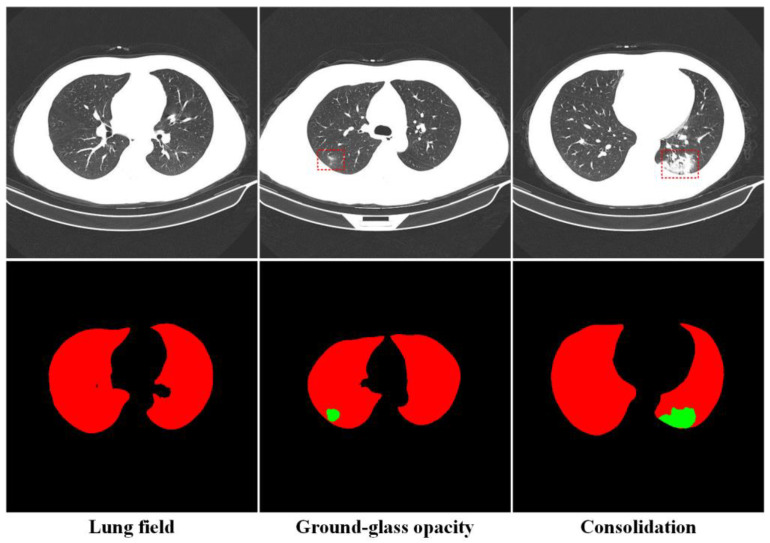
Sample images of lung field, ground-glass opacity, and consolidation in CT images.

**Figure 3:**
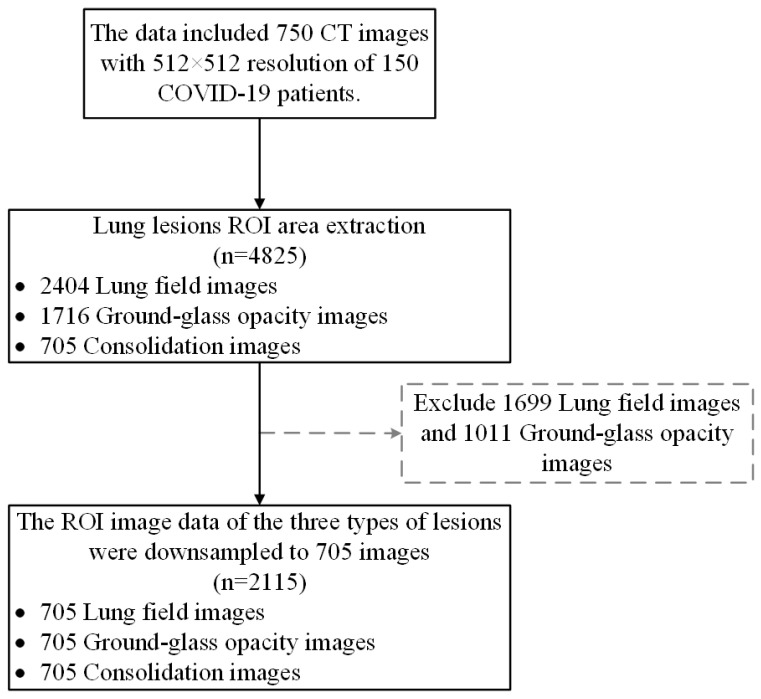
Flow chart of data inclusion and exclusion criteria.

The dataset includes two parts: the lung disease classification data and the segmentation data. The classification data involve lung-CT images of novel coronavirus pneumonia and common pneumonia, as well as a normal control group, and the corresponding clinical diagnosis data. The lesion segmentation data were obtained from the CT slice images of CC-CCII. The data included 750 CT images, with 512 × 512 resolution, of 150 COVID-19 patients. Each image was manually segmented into background, lung field (LF), ground-glass opacity (GGO), and consolidation (CL). Lung imaging manual annotation was performed by eight radiologists; four of the radiologists have 5 to 15 years of clinical experience, and four of the radiologists have 15 to 25 years of clinical experience. In cases involving disputes, a final consensus was reached by an independent panel of four senior radiologists, each with at least 25 years of clinical experience. Figure 2 shows a sample of CT images of lung fields, ground-glass opacity and consolidation. In this paper, the CC-CCII segmentation dataset is used as the research object. In the process of image preprocessing, the ROI region for lesions larger than 9 × 9 was selected. The data include 2404 lung field images, 1716 ground-glass opacity images, and 705 consolidation images. Due to the imbalance of the data, which affects the performance of the model, the ROI image data of the three types of lesions were downsampled to 705 images, resulting in a total of 2115 case data, for the subsequent construction of the deep learning and radiomics joint model. Figure 3 shows a flow chart of inclusion and exclusion criteria.

### 2.2. Radiomics Feature Extraction

**Figure 4:**
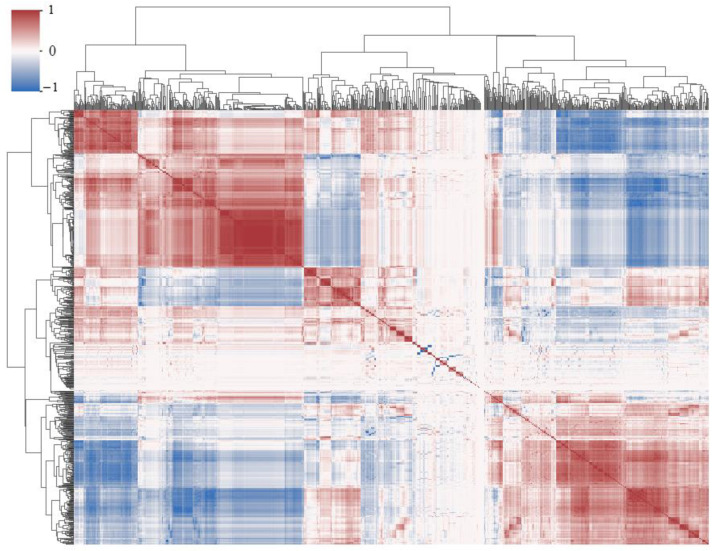
Cluster plot of radiomics feature correlation.

**Figure 5:**
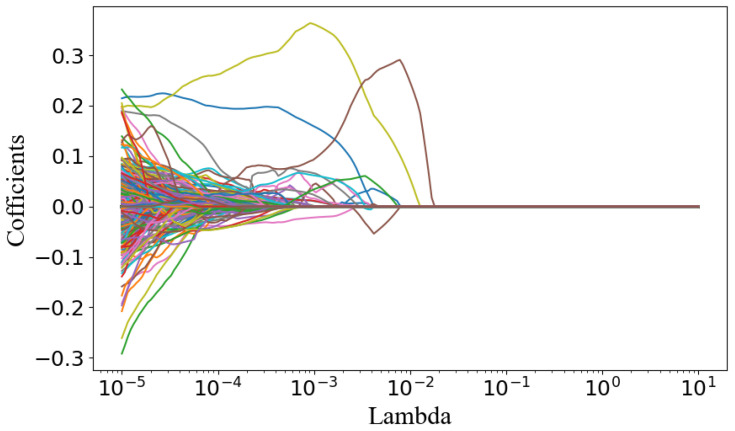
Lambda curves for LASSO regression coefficients.

**Figure 6:**
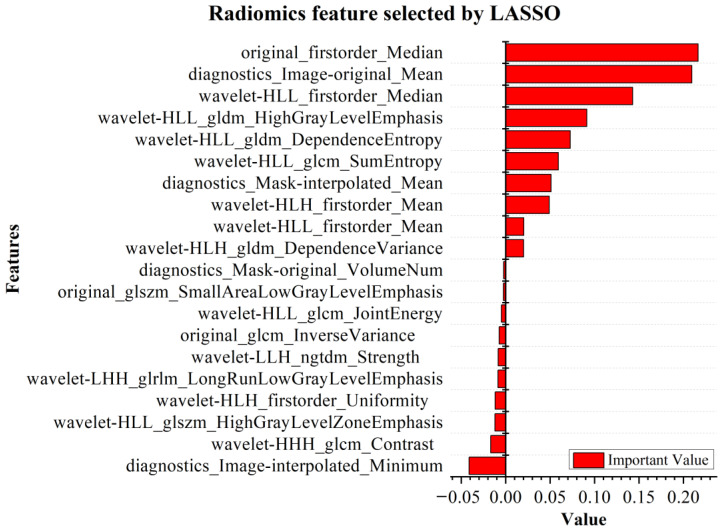
The 20 dimensional key features, as selected by radiomics.

Radiomics can extract a large number of high-dimensional image information elements, which permits the analysis of disease information in a more objective and comprehensive manner, and plays a potential role in promoting disease diagnosis, treatment selection and prognosis evaluation [27]. In this paper, the Pyradiomics package (version 3.1.0) is used to extract 873 dimensional radiomics features of lung lesions. The extracted features mainly include first-order statistical features, shape features, gray-level co-occurrence features, gray-level dependence matrix features, gray-level run-length matrix features, gray-level size zone matrix features, and neighboring gray-tone difference matrix features of original images and wavelet transform images. The correlation clustering plot is shown in Figure 4, and reveals that the majority of radiomics features exhibit both correlation and redundancy. Therefore, the least absolute shrinkage and selection operator (LASSO) [28] was used to select features from high-dimensional radiomics data, and the top 20 key features were selected as the features of the model input. The Lambda curve of regression coefficient in the feature selection process of LASSO regression model is shown in Figure 5, and the selected key radiomics features and their importance are shown in Figure 6.

According to the results of LASSO feature selection, the first five key characteristics are “original firstorder Median”, “diagnostics Image-original Mean”, “wavelet-HLL firstorder Median”, “wavelet-HLL gldm High Gray Level Emphasis”, and “wavelet-HLL gldm Dependence Entropy”. Among them, the original firstorder Median feature has the highest impact on the prediction target. This feature can describe the average brightness distribution of the original-image gray value and measure the central trend of image brightness value, and can be used to evaluate the disease state or potential biological characteristics, such as tumor texture and tissue density. In addition, the remaining four importance degrees mainly represent the overall brightness of the original image, the local texture feature intensity, the texture feature of the high gray-level region, and the uncertainty or randomness of the gray-level information between pixels.

### 2.3. Deep Feature Extraction

**Figure 7:**
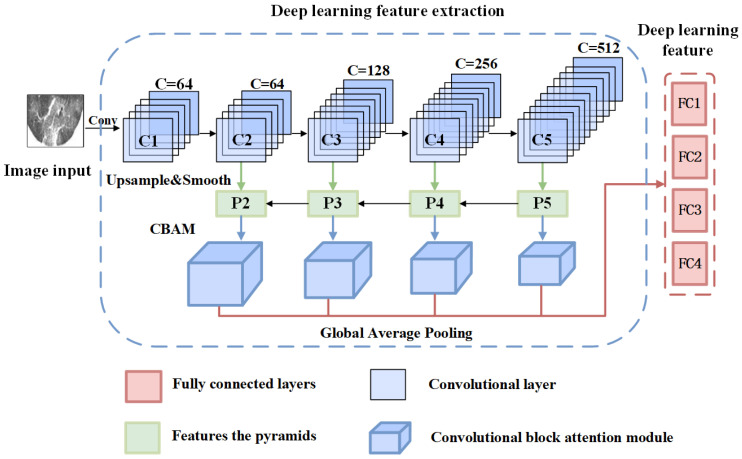
MFPN neural network structure diagram. C represents the convolutional layer, P represents the pyramid feature, and FC represents the fully connected layer.

In this paper, we use the deep learning framework Pytorch (version 1.4.0) to construct a new Multi-Feature Pyramid Network (MFPN) to extract high-dimensional deep features of lung lesions. This model uses ResNet34 as the baseline network, and uses Feature Pyramid Networks (FPN) [29], convolutional attention, global average pooling and other means to build the classification model. It can effectively extract the channel features and spatial features of different scales, and effectively solve the problem of insufficient semantic information extraction in the process of feature extraction. The structure diagram of the MFPN model is shown in Figure 7. In this study, the fully connected layers FC1, FC2, FC3, and FC4 of the last layer of the MFPN network are used as the final deep features.

**Figure 8:**
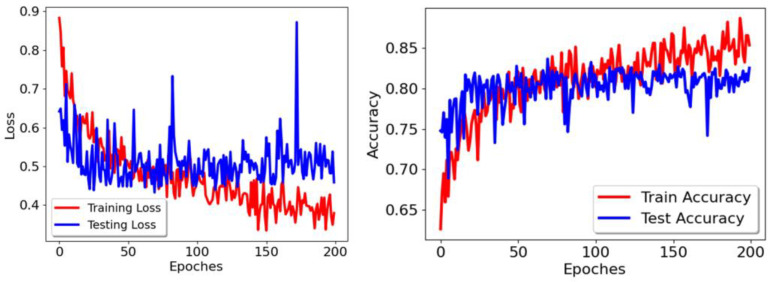
Loss function and accuracy curves of the MFPN network.

In addition, in order to ensure that the hyperparameters of the neural network can converge to the optimal state, the training epoch is 200 rounds, the batchsize is 8, and the loss function uses the cross-entropy loss. In order to avoid the scenario of the loss function falling into the local optimal solution, which would result in the model performance not reaching its optimal effect, Adam is selected as the optimization method. The initial learning rate is 0.0001. The data augmentation methods used include random cropping, flip transformation, and scaling. The changes of loss function and accuracy when the MFPN network is trained are shown in Figure 8. When the model is trained to 70 epochs, the accuracy of the model on the test set is the highest, reaching 83.63%.

### 2.4. Feature Fusion and Modeling

Multi-feature fusion is often helpful to improve model performance, increase feature diversity, and alleviate overfitting. Therefore, in order to further discover the regularity of imaging features of lung disease lesions and verify the influence of multi-omics data on lung diseases, this paper combines the deep features of lung lesions and radiomics features; the deep features use the 5-dimensional features of FC1, FC2, FC3, and FC4 in the MFPN model, and a total of 20 dimensional deep features. The radiomics features are the 20 dimensional features selected by LASSO. Finally, the classification models are Logistic, KNN, Bayesian, Random Forest, XGBoost, and Deep Learning; these six machine-learning methods were used to construct multi-classification models for distinguishing the typical types of lung lesions.

## 3. Results

In this paper, 20 dimensional deep features and 20 dimensional key radiomics features are used to quantitatively analyze the performance of machine-learning models in lung lesion classification. In the experiment, the classification performance levels of radiomics, deep features and combined radiomics features and deep features are compared and analyzed, respectively. The classification models are Logistic, KNN, Bayesian, Random Forest, XGBoost, and Deep Learning; these six models are used to quantitatively compare and analyze the classification performance. The deep model not only uses the deep features combined with other machine-learning classification methods, but also uses the traditional deep feature classification method for modeling. The results of the comparative experiments are shown in Table 1.

In the experiment, six methods were used to evaluate the classification effect under the three combined features. It can be seen from Table 1 that when the 20 dimensional key radiomics features were used for modeling, the Logistic achieved the best classification effect, and its classification accuracy was 90.07%. The classification accuracy levels of KNN, Bayesian, Random Forest, and XGBoost were 89.60%, 86.76%, 89.36%, and 88.89%, respectively. Random Forest and the KNN model also show good performance in radiomics features-based modeling. In addition, 20 dimensional deep features extracted by deep learning were used for experiments; the classification accuracy levels of Logistic, KNN, Bayesian, Random Forest, XGBoost, and Deep Learning were 69.50%, 72.34%, 46.81%, 74.23%, 75.41%, and 83.63%, respectively. Among them, the classification method using traditional deep learning has the best performance. Finally, the key radiomics features and deep features were combined for experiments; the experimental results show that XGBoost has the best classification performance, and its classification accuracy is 89.83%. The accuracy of Logistic, KNN, Bayesian, and Random Forest were 87.00%, 84.40%, 82.74%, and 89.36%, respectively. According to the results, the key radiomics features and the combination features have high classification performance, and Logistic has the best classification performance for the key radiomics features; its classification accuracy can reach 90.07%.

To further evaluate the performance of the model, the two models with the highest classification accuracy, Logistic and XGBoost, were selected, and each lesion subtype was evaluated in detail using Precision, Recall, F1-Score, and AUC metrics. Table 2 shows the detailed evaluation metrics for Logistic and XGBoost, respectively.

**Figure 9:**
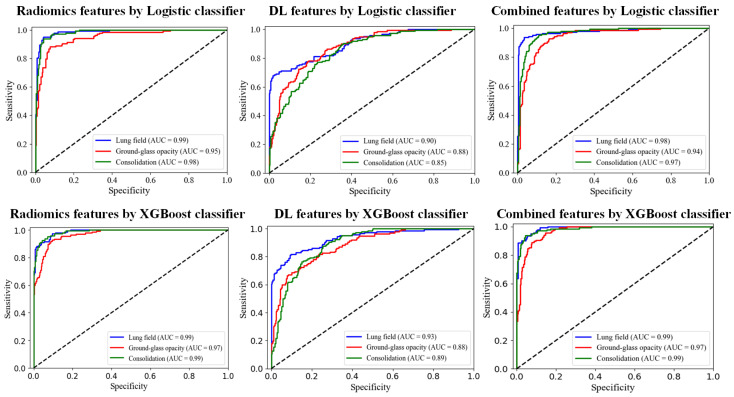
ROC curves for Logistic and XGBoost models.

According to Table 2, Logistic performs best in the key radiomics features, with an average precision of 90.04%, an average recall of 90.14%, an average F1 value of 90.06%, and an average AUC value of 97.32%. In addition, the average AUC values when using deep features and joint features in the Logistic model are 87.49% and 96.19%, respectively. Logistic achieved the highest AUC value for the key radiomics features, and its corresponding performance is also the best. The average AUC value of XGBoost in key radiomics features was 98.13%, and the average AUC value of XGBoost in deep features was 89.94%. In the joint feature, XGBoost has the best comprehensive performance, with an average precision of 88.91%, an average recall of 90.93%, an average F1 value of 89.78%, and an average AUC value of 98.08%. For different types of lung lesions, the XGBoost model performed better in the combined features than the key radiomics features in Lung field and Consolidation lesion types, with slightly lower Recall and F1 values in Ground-glass opacity. The ROC curves of Logistic and XGBoost under the three categories are shown in Figure 9.

## 4. Discussion

In this study, deep learning and radiomics methods were used to construct an image classification model for lung lesion subtypes of COVID-19. The proposed method can provide an automatic classification method for the diagnosis of lung lesions, one which can be used to solve the problem of difficulty in the diagnoses of a large number of lung lesions. The proposed model can be used to distinguish Lung field, Ground-glass opacity and Consolidation in CT images of COVID-19 patients, and radiomics was used to analyze the imaging differences of lung lesions. Experimental results using six classification models show that the highest classification accuracy values for key radiomics features, deep learning, and combined features are 90.07%, 75.41%, and 89.83%, respectively, and the classification accuracy of the traditional deep learning method is 83.63%. It can be seen from the results that the performance of the model constructed by radiomics features is better than that from the depth features. The main reason is that the ROI regions of lung lesions used in the dataset of this study are quite different, showing that the size of lesions is not uniform. And a deep learning model of uniform image patterns can show better performance in the task, while lesion size differences can reduce the performance of the model [30,31]. However, for combined features, the robustness of the model is improved.

Among them, the Logistic model has the best performance in key radiomics features, the traditional deep learning method has the best performance in deep features, and XGBoost has the best performance in joint features. Moreover, with the combination of the key radiomics and deep features, the complexity and dimension of the data increase, the performance levels of the Logistic, KNN, and Bayesian models show a trend of decrease, and the classification performance levels of XGBoost and Random Forest are improved. Therefore, XGBoost and Random Forest have better generalization performance when the data complexity increases and the data dimension increases. Moreover, according to Table 1, the classification accuracy of the deep features is significantly lower than those of the key radiomics features and the joint features. In the case of Logistic model, the joint feature modeling method does not improve the performance of the model, but rather decreases it. However, the joint features under the XGBoost model can effectively improve the classification accuracy of the model. Therefore, it can be seen that in the classification task of lung lesion images, the joint feature composed of deep features and radiomics features does not improve the classification performance of simple models, but does have a certain positive effect on models with a strong fitting ability. This further proves that the ensemble learning approach can show excellent performance in complex tasks [32].

In order to further illustrate the robustness of the model, different categories are usually evaluated by additional evaluation indicators [33]. For three different subtypes of COVID-19 lung, the two models with the highest classification accuracy, Logistic and XGBoost, were selected for comparison experiments. It was found that the use of key radiomics features in Logistic could achieve the highest classification performance in the three subtypes, with an average AUC value of 97.32%. In this model, the indices of the Lung field subtype were significantly higher than those for Ground-glass opacity and Consolidation, and the indices of Consolidation subtype were significantly higher than those for Ground-glass opacity. This indicates that the Ground-glass opacity subtype has imaging features which are less obvious than those of Lung field and Consolidation. In addition, using the combined features in XGBoost can achieve a good level of performance in the Lung field and Consolidation subtypes; the accuracy of the Ground-glass opacity subtype is higher than those found with key radiomics features and depth features, and the other indicators are not much different. The average AUC values of XGBoost in key radiomics feature modeling and joint feature modeling are 98.13% and 98.08%, respectively. Although the AUC of the key radiomics feature modeling is slightly higher than that of the joint feature, the comprehensive performance of joint feature modeling is significantly better than that of key radiomics. Therefore, in the task of lesion recognition in lung images of COVID-19, the joint modeling method can improve the recognition performance and generalization ability, and is suitable for large-scale and high-dimensional radiomics analysis tasks. In addition, radiomics features were used to analyze the differences in imaging phenotypes associated with different lung lesions. Through the selection of radiomics features, it was determined that that the original firstorder Median feature has the highest impact on the prediction target. The results showed that for Lung field, Ground-glass opacity, and Consolidation, the three principal differences within Lung lesions, the differences are mainly reflected in the aspect of image brightness. In addition, several other important radiomics features also indicated that the randomness of local texture feature intensity and gray-level information was also an important imaging marker affecting the difference of lung lesions.

## 5. Conclusions

In this study, deep learning and radiomics methods are used to distinguish different lesion types in COVID-19 images, and the MFPN model is proposed as a means to extract the depth features of lesions; the classification performance levels of six common machine-learning methods are subsequently compared. The experimental results show that in the COVID-19 image classification task, the classification method combining radiomics and deep features can achieve good classification results and has certain clinical application value. In addition, we analyzed the differences in the imaging of phenotypes of different lung lesions by radiomics features, which provided a reference for the imaging identification of lung lesions.

## Figures and Tables

**Table 1:** Model comparison using the experimental results, as evaluated for accuracy.

Model	Radiomics	Deep Learning	Combined Feature
Logistic	90.07%	69.50%	87.00%
KNN	89.60%	72.34%	84.40%
Bayesian	86.76%	46.81%	82.74%
Random Forest	89.36%	74.23%	89.36%
XGBoost	88.89%	75.41%	89.83%
Deep Learning	-	83.63%	-

**Table 2:** Classification results for the Logistic and XGBoost models.

Data Type	Metrics (%)	Logistic Model				XGBoost Model			
		LF	GGO	CL	Mean	LF	GGO	CL	Mean
Radiomics	Precision	92.25	87.59	90.28	90.04	89.44	88.32	87.5	88.42
	Recall	93.57	83.33	93.53	90.14	92.03	85.21	94.03	90.42
	F1-Score	92.91	85.41	91.87	90.06	90.71	86.74	90.65	89.37
	AUC	98.63	94.98	98.35	97.32	98.78	96.87	98.74	98.13
Deep learning	Precision	68.31	78.83	61.81	69.65	77.46	68.61	62.5	69.52
	Recall	90.65	58.7	67.42	72.26	83.33	74.02	76.27	77.87
	F1-Score	77.91	67.29	64.49	69.9	80.29	71.21	68.7	73.4
	AUC	89.95	87.55	84.96	87.49	92.96	88	88.85	89.94
Combined feature	Precision	92.25	83.94	84.72	86.97	89.44	89.78	87.5	88.91
	Recall	92.25	79.31	89.71	87.09	94.78	82.55	95.45	90.93
	F1-Score	92.25	81.56	87.14	86.98	92.03	86.01	91.3	89.78
	AUC	97.79	94.08	96.7	96.19	98.96	96.74	98.53	98.08

## Data Availability

The data is from the China National Center for Bioinformation, via the China Consortium of Chest CT Image Investigation (CC-CCII). It is publicly available globally at http://ncov-ai.big.ac.cn/download (accessed on 18 June 2024).
